# Where Is the Honey Bee Queen Flying? The Original Case of a Foraging Queen

**DOI:** 10.3390/insects12111035

**Published:** 2021-11-17

**Authors:** Ignazio Floris, Michelina Pusceddu, Pietro Niolu, Alberto Satta

**Affiliations:** 1 Department of Agricultural Sciences, University of Sassari, Viale Italia 39A, 07100 Sassari, Italy; mpusceddu@uniss.it (M.P.); albsatta@uniss.it (A.S.); 2 Independent Researcher, Via Sassari 130, 07041 Alghero, Italy; aniolu@uniss.it

**Keywords:** queen bee, *Apis mellifera*, foraging activity, feeding behaviour

## Abstract

**Simple Summary:**

Reproduction is the only task normally attributed to the queen due to its specific morpho-functional characteristics, while foraging activities are exclusively carried out by workers bees in the honey bee colony. For example, the queen’s proboscis is shorter than that of workers and, therefore, less suitable for exploring the inside of flowers to collect nectar. Olfactory and visual detection is also less developed in the queen than in workers, and it is well known how important these stimuli are in order to search for appropriate flowers and find the food source within the flower. In the countryside of northern Sardinia, a honey bee queen (*Apis mellifera* L.) was detected for the first time while foraging on a flower (a borage flower), most likely during an orientation flight before mating. The open, shallow corolla, and the excellent nectar secretion of the borage flower might have facilitated the queen bee activity. This new queen behaviour was based on the morphological traits of the specimen collected and photos taken in that moment. The observed foraging activity opens new and yet unexplored perspectives on the behaviour of queen bees outside the nest (or the hive), which could occasionally include tasks usually attributed only to workers.

**Abstract:**

During a bee fauna survey in the countryside of northern Sardinia, a honey bee queen (*Apis mellifera* L.) was detected while foraging on a borage (*Borago officinalis* L.) flower in Uri, Province of Sassari, Italy, most likely during an orientation flight before mating. Morphological details, detectable from photos with the naked eye and stereomicroscopic observations, confirmed that the honey bee queen was sucking nectar from a flower. The enormous development of the abdomen, lack of pollen-collecting structures in the legs and other characteristics such as the typical distally bilobed shape of the mandibles, with long hairs on their outer surface, proved the structural differences between the queen specimen and the other castes of bees. The queen’s proboscis, which is shorter compared to the workers, may have been counterbalanced by the shape and nectar production of the borage flower. This new observation proves that the queen can feed herself under natural conditions, likely to obtain the energy required for flying. Although we cannot exclude disturbing factors that could explain this foraging behaviour of a queen observed for the first time, this note opens a new scenario and discusses this new finding in the context of the available literature on the queen’s behaviour and questions to be answered.

## 1. Can the Honey Bee Queen Forage from Flowers?

**Figure 1:**
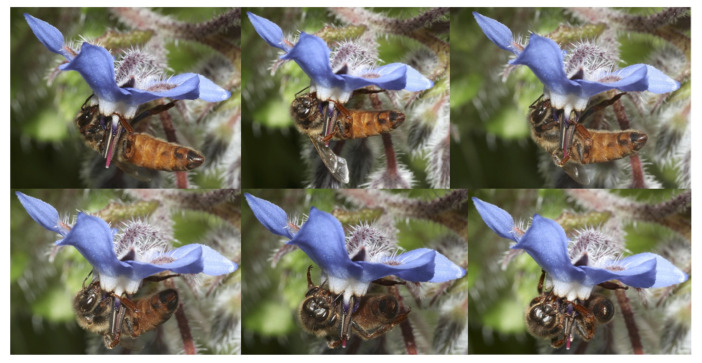
A honey bee queen (*Apis mellifera* L.) while feeding on a borage flower (*Borago officinalis* L.) (Uri, Sardinia, Italy; photo: Pietro Niolu).

**Figure 2:**
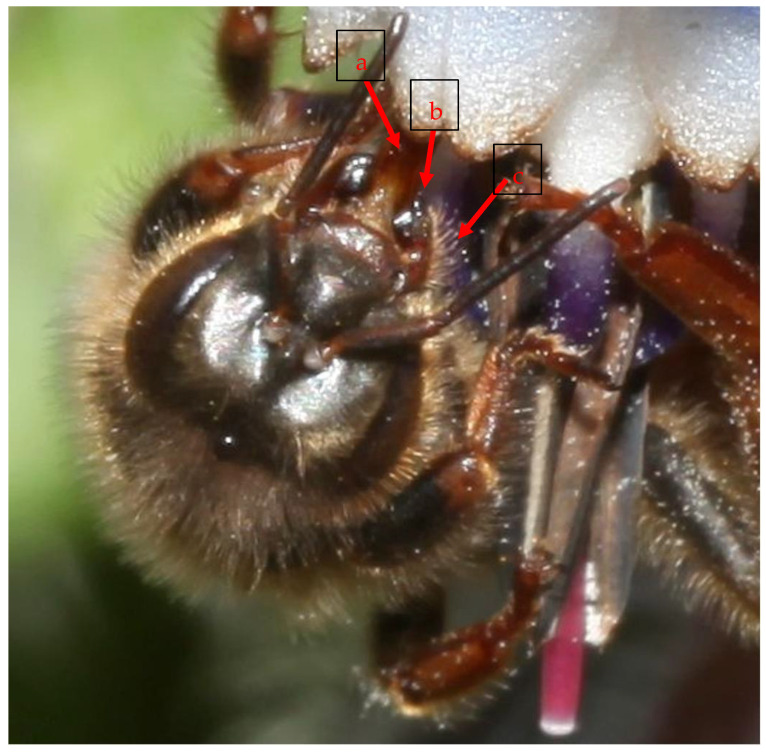
Morphological details of the mouthparts of the observed queen bee: (**a**) extended proboscis; (**b**) bilobed shape of the mandible with the outer lobe toothlike; (**c**) long hairs on the outer surface of the mandibles (Uri, Sardinia, Italy; photo: Pietro Niolu).

Figure 1 and Figure 2 seem to provide an affirmative answer to this question. We observed a honey bee queen (*Apis mellifera* L.) foraging on a flower in the field for the first time. The observed behaviour suggests that the queen can feed herself under natural conditions, probably to obtain the energy needed for flying.

This new observation was made during a survey on bee fauna in Sardinia (Uri, Province of Sassari, Italy) on 23 April 2021, at 12:58. In a monitoring session, a honey bee queen was detected with naked-eye observation followed by a sequence of photos while feeding on a borage (*Borago officinalis* L.) flower, and was then captured. Observing the details of the photos (Figure 2) and of the sampled specimen, under the stereomicroscope, some characteristic morphological traits of the honey bee queen were confirmed, such as the evident extended proboscis for collecting nectar. Although the queen’s proboscis is shorter than that of the worker [1], it could be suitable for sucking nectar from the borage flower thanks to the open, shallow corolla, and the excellent nectar secretion of this flower [2]. Other visible peculiarities observed were the typical distally bilobed shape of the mandibles, with the outer lobe being a long toothlike projection, the presence of long hairs in the outer surface of the mandibles, and the enormously developed abdomen [1].

Before our finding, the previous literature [3,4] had not reported a honey bee queen feeding herself outside the hive as foraging workers normally do.

## 2. Are the Morpho-Functional and Biological Characteristics of the Queen Suitable for Foraging on Flowers?

It is well known that honey bees are eusocial insects characterized by three castes (queen and workers from fertilized eggs and drones from unfertilized ones), with a reproductive division of labour in the colony, performed mainly by workers according to a sequence of activities [3,5]. Among these activities, foraging is an exclusive task of the workers and is normally performed in the last period of their life. It consists of collecting nectar, honeydew, pollen, water or resins to satisfy the needs of the colony. Unlike the workers, the queen does not perform various jobs during its life, also due to the specific morpho-functional differences between bee castes. For example, the queen proboscis is shorter than that of workers and, therefore, less suitable for exploring the inside of flowers to collect nectar. Indeed, in honey bee colonies, adult queens are normally fed by workers, mostly with brood food and often some additional honey, and hardly ever feed themselves. For instance, under experimental conditions, isolated queens fed on sugar candy and survived for many weeks [3]. Compared with workers, the honey bee queen also lacks pollen-collecting structures in the legs and has a larger and less slender body, due to the enormously developed ovaries and abdomen, with an egg-laying function. All these typical characteristics of the queen allow us to easily recognise her from the other castes, as was achieved in this study.

From a biological point of view, it is important to highlight that the worker honey bees need to train themselves by participating in pre-foraging flights before becoming foragers [6] and, similarly, queens perform orientation flights before participating in mating flights [7,8].

The pre-foraging stage for workers, as the pre-mating stage for the queens, represents a critical phase which includes high mortality risks due to environmental hazards (e.g., pollution, parasites, climate and predation) [6,9]. However, this stage is fundamental because it allows bees to acquire cognitive capacities essential for their future performance, such as navigation and homing [8,10,11].

Orientation flights may also be crucial for the survival of virgin queens. Adequate nutritional conditions may help to improve flight performance and overcome possible threats. This could explain the queen’s need to forage on flowers during her orientation or mating flights. What is surprising is that queen bees are able to do it in the same way the worker bees do.

## 3. Is the Queen Able to Perceive the Scent and Visual Cues of Flowers for Foraging?

Among the morpho-functional traits involved in foraging activity, the sensory structures of honey bees play a fundamental role. Although the olfaction of the queen has received less attention compared to that in drones and workers [12], there is no doubt that the perception of olfactory and visual stimuli is required for visiting flowers.

The placoid olfactory sensilla occupy most of the surface area of the antenna. Most of the placoid receptor cells respond to several compounds (generalist cells) and can be stim-ulated by a range of plant volatiles compounds in bees. However, because olfactory studies have focused mainly on the detection of sex and aggregation pheromones rather than on ordinary odours [13], little is known about how honey bee sensory neurons detect non-pheromonal substances. Another interesting aspect concerns the different characteristics of the olfactory receptors of the antennal sensilla between castes of bees. For example, a worker’s antennae show a higher expression of 67 Or genes, which are involved in pheromone communication and in the perception of cuticular hydrocarbons and of floral scents [12]. In contrast, drone antennae show a higher expression of 24 Or genes, including the receptor for the major queen pheromone compound 9-ODA, which plays an important role in sex-pheromone communication [12]. Similar studies should be conducted in queen bees to better understand gene expression in this caste and clarify to what extent they are able to perceive odours from flowers.

It is well known that the bee must collect its food at some distance from the nest (or the hive) by relying largely on visual cues to navigate to the foraging sites. Once the bee reaches the flowers, visual cues are needed to select appropriate flowers and to find the food source within the flower. For this kind of foraging behaviour to be successful, the bee must have sensory equipment to distinguish between flower species, based on the size, shape, colour and plane symmetry of the individual flowers, in addition to their scent [14]. Because the compound eye of the queen includes a lower number of ommatidiae (3500) compared to a worker (5000–6000) or a drone (10,000) eye [1], it is likely that queen bees are less able to perceive visual stimuli. Based on our observation of a queen bee foraging on an open borage flower, it seems that the queen can identify flowers visually. However, given the accidental nature of our finding, the question remains open, and in order to better understand this queen behaviour outside the hive, more detailed investigations will be needed.

## 4. What Is the Behaviour of Drones during Their Flights?

**Figure 3:**
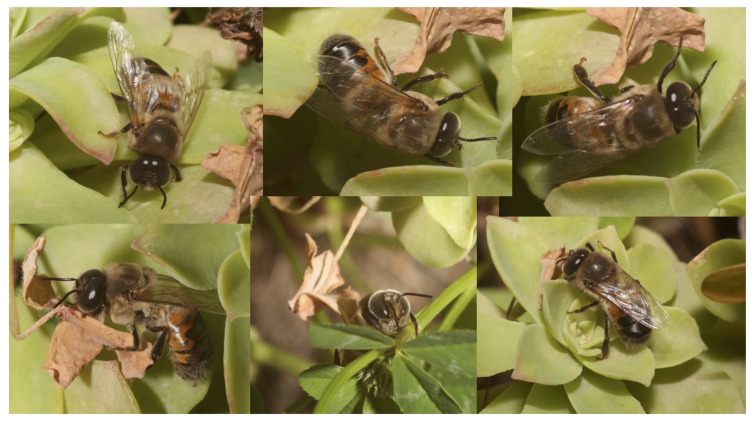
A honey bee drone (*Apis mellifera* L.) on an *Aeonium* sp. plant (Crassulaceace) (Uri, Sardinia, Italy; photo: Pietro Niolu).

Another type of additional information that needs to be gathered concerns drone flight. In our survey, we also detected the presence of drones on plants during the day (Figure 3). However, we do not know whether the drones rested on a plant during an orientation flight, while looking for food, or due to disorientation caused by pathogens or pesticides, which may affect drone scouting activity.

Recently, the activity of *A. mellifera* drones was monitored during their entire life in spring and summer using an optical bee counter placed at the entrance of the hive [15]. Drones were active in the afternoon, and most flights occurred from 14:00 to 18:00. Short orientation flights occurred when drones were 6–9 days old, and mating flights of about 30 min occurred from 21 days of age on in spring and from 13 days of age on in summer [15]. Drones, similarly to the queens, do not collect food, but when stimulated by workers through vibration signals [16,17], they can perform trophallaxis. Moreover, drones can participate in the thermoregulation of the nest by heat production [18,19]. Before reaching sexual maturity, drones can also make orientation flights around the nest [7,20], usually in the afternoon, to learn the features of the landscape that allow them to go back to the nest [8,21,22].

When drone activity was monitored for the first time during the whole day in northwest Argentina, several drones were active not only in the afternoon, as previously reported, but also in the morning and late morning. It was hypothesized that the morning activity of drones could be due to drifting, possibly when looking for food, or due to the presence of hives containing queen cells that attracted nearby drones [23].

Considering that the queen and drones have an almost exclusive specialized function in the colony associated with reproduction, a better understanding of the behaviour of drones outside the hive before mating could be helpful to elucidate the queen’s behaviour.

## 5. What Is the Behaviour of Queen Bees during Their Orientation Flights?

Honey bee queens perform a few short orientation flights before the nuptial mating flights [7,8]. Mating takes place in specific sites, known as drone congregation areas [12,14,15], where thousands of drones congregate at certain times of the day [18,19]. A queen can mate various times during one flight and can perform several consecutive nuptial flights in one day or various days [7,21,24,25,26]. The number of drones a queen mates with is on average 12–14, ranging from 6 to 26 [27,28,29,30]. However, queen behaviour during free flight remains largely unknown. Apart from the great variations observed in the return frequency and flight duration of released queens during their flights [24], no other reports on such behaviour have been published.

The effects of the vibratory dance on the activities of virgin queens was investigated. It is known that virgin queens are forced by the workers to fly, often in the mid-afternoon or afternoon, and most of them take one or two orientation flights and from one to five mating flights in two to four days [4,16]. Based on various studies [25,26,27,31,32,33], the distance between mating sites and the source colonies of drones and queens is normally between 5 and 7 km, but can vary from 2 km to a maximum of about 17 km.

Because flying is costly in terms of energy expenditures [28], we can assume that the queen, while undertaking flights that are often very distant from the native hive or the congregation sites, may need to feed during the trip.

## 6. Does the Queen Visit Flowers to Supply Herself with Sugars during Her Flights?

The queen foraging behaviour we observed on a flower for the first time may be explained by the pre-mating biology of the queen. In the pre-mating period, the newly emerged queen moves around in the brood area to destroy sealed queen cells, which would be potential competitors. Soon afterwards, the workers show an antagonistic behaviour towards the queen by pushing and biting the virgin queen and sometimes using “balling” behaviour against her. This forces the queen to fight back by producing sounds and vibrations that force the workers to stop this hostility. The peak of this aggressive behaviour occurs in the early afternoon, coinciding with the orientation or mating flights of the queens [4,24,30]. Therefore, it is possible or likely that the queen, after suffering the energetic stress required to oppose the workers, may seek a suitable source of food during her orientation or mating flight to replenish the energy reserves that are necessary to continue flying. Another scenario could be that, when the queen is surprised by cold weather during a mating flight, she may need to forage.

## 7. Final Remarks

There is no doubt that we accidentally observed a queen bee sucking nectar on a borage flower. This most likely occurred during an orientation or mating flight, although we cannot exclude other possible disturbing factors. Certainly, the observed foraging behaviour needs to be confirmed by other observations and further investigated, e.g., by using advanced technology such as the most promising remote sensing methods [29]. This would improve the poor knowledge on the behaviour of newly emerged queen bees and would help us better understand their biology, apart from their already known fundamental role in honey bee colonies after mating.

## Data Availability

The data presented in this study are available on request from the corresponding author.
